# Heat stress reveals bidirectional cross talk between the heat shock response and UPR^ER^ in *C. elegans*

**DOI:** 10.1093/g3journal/jkag141

**Published:** 2026-06-22

**Authors:** Athena Alcala, Toni Castro Torres, Rebecca Aviles Barahona, Phillip A Frankino, Ryo Higuchi-Sanabria, Gilberto Garcia

**Affiliations:** Leonard Davis School of Gerontology, University of Southern California, Los Angeles, CA 90089, United States; Leonard Davis School of Gerontology, University of Southern California, Los Angeles, CA 90089, United States; Leonard Davis School of Gerontology, University of Southern California, Los Angeles, CA 90089, United States; Department of Molecular & Cell Biology, Howard Hughes Medical Institute, University of California, Berkeley, Berkeley, CA 94720, United States; Leonard Davis School of Gerontology, University of Southern California, Los Angeles, CA 90089, United States; Leonard Davis School of Gerontology, University of Southern California, Los Angeles, CA 90089, United States

**Keywords:** heat stress, endoplasmic reticulum unfolded protein response, heat-shock response

## Abstract

Organisms rely on coordinated stress responses to maintain cellular homeostasis. Perhaps the best-known example of multiple stress inputs converging onto a single response is the integrated stress response, which reduces global translation under various stressed conditions to reduce the protein folding burden of the cell. Similarly, most stress responses generally involve coordination of additional protein homeostasis (proteostasis) pathways, including increased expression of chaperones to refold proteins, as well as activation of clearance mechanisms, such as autophagy and the ubiquitin proteasome system. Our study investigates how heat stress can influence coordinated activation of both cytosolic and endoplasmic reticulum (ER) chaperones, exploring bidirectional cross talk between canonical activators of the cytosolic heat-shock response (HSR) and the unfolded protein response of the ER (UPR^ER^). Using robust transcriptional reporters in the *Caenorhabditis elegans* model system, we explore a noncanonical activation of the UPR^ER^ under heat stress by the coordinated effects of XBP-1 and HSF-1. We further investigate inter-tissue communications of stress whereby neuronal or glial activation of the UPR^ER^ can result in heterotypic enhancement of the HSR in peripheral cells and can increase thermotolerance. This work highlights the complex convergence of cellular stress responses, a phenomenon that may reflect a general strategy wherein localized stress can activate numerous proteostasis pathways to prevent whole-cell and whole-organism damage.

## Introduction

Organisms are constantly exposed to stress, which can have detrimental effects on health and physiology. As such, several regulatory stress responses function to promote and maintain cellular homeostasis, tailored for each specific organelle. For example, the unfolded protein response of the endoplasmic reticulum (UPR^ER^) is activated under endoplasmic reticulum (ER) stress by unconventional splicing of *xbp-1* mRNA by the ER-resident transmembrane protein, IRE-1. This results in translation of functional XBP-1s to activate transcription of genes essential for ER function (Walter and Ron 2011; Frakes and Dillin 2017). In addition to this most well-studied IRE-1/XBP-1 arm, 2 additional branches of UPR^ER^ are mediated by protein kinase R-like endoplasmic reticulum kinase (PERK, or PEK-1 in *Caenorhabditis elegans*) and activating transcription factor 6 (ATF-6). Similar to IRE-1, PEK-1, and ATF-6 are ER-resident transmembrane proteins containing a luminal domain that can bind unfolded proteins in the ER (Schröder and Kaufman 2005). Under ER stress, PEK-1 activation results in phosphorylation of eukaryotic initiation factor (EIF-2A) to reduce global translation and reduce the protein folding burden of the ER (Ma and Hendershot 2003; B’chir et al. 2013; Han et al. 2013). ATF-6 activation involves cleavage of the cytoplasmic domain and translocation into the Golgi for further processing (Haze et al. 1999; Ye et al. 2000).

A similar machinery, the heat-shock response (HSR), exists for the cytosol, which is regulated by heat-shock factor 1, HSF-1. Under normal, nonstressed conditions, HSF-1 is maintained in an inactive form by direct binding with HSP-70 and HSP-90, which act as negative regulators of HSF-1 function (Shi et al. 1998; Anckar and Sistonen 2011; Neef et al. 2014). In response to stress, these molecular chaperones are titrated away from HSF-1, allowing for a series of posttranslational modifications that promote trimerization and nuclear translocation of HSF-1, allowing for transcriptional activation of HSR targets (Anckar and Sistonen 2011; Dai 2018).

Much of the foundational research in stress biology has understandably used a reductionist approach studying individual stress response pathways within specific organelles, enabling careful dissection of their molecular mechanisms. However, growing evidence suggests that stress responses often have pleiotropic effects, influencing multiple organelles and systems, highlighting the need to also consider inter-organelle communication and systemic coordination of stress. For example, low levels of mitochondrial stress can increase functional capacity of the HSR, delaying age-associated loss of HSR function to promote stress resilience and longevity (Labbadia et al. 2017). In addition, knockdown of the *hsp-6*, which encodes a mitochondrial chaperone, resulted in activation of the cytosolic HSR through a complex signaling cascade involving fatty acid remodeling (Kim et al. 2016). Finally, HSF-1 impacts mtDNA gene expression by elevating histone H4 levels and altering mtDNA chromatin state, which can activate the unfolded protein response of the mitochondria (UPR^MT^) to promote longevity (Sural et al. 2020).

Similar overlapping functions of the UPR^ER^ and HSR have also been observed. One study found 9 stress-responsive genes overlapping under ER stress and heat stress conditions (Liu and Chang 2008), including the ER chaperone, HSP-4/BiP (Kohno et al. 1993), and the COPII cargo receptor, Erv29 (Caldwell et al. 2001). In addition, activating the HSR through a constitutively active form of HSF-1 has been shown to compensate for growth defects in cells lacking a functional UPR^ER^ (Liu and Chang 2008). Heat-shock has also been shown in mammalian cells to activate canonical XBP1 targets (Heldens et al. 2011). These results are not surprising considering the pleiotropic effects of heat stress exposure: exposure to heat would likely result in protein unfolding in all cellular compartments and not just the cytosol, thus likely requiring activation of multiple stress response pathways. However, whether heat-shock can directly activate UPR^ER^ in *C. elegans* and what specific stress response pathways are required for this activation have yet to be studied. One study demonstrated that *C. elegans* electrotaxis behavior is regulated by interconnected cytosolic, mitochondrial, and ER stress response pathways, including the HSR and UPR, further suggesting an overlapping and coordinated nature of these stress responses (Taylor et al. 2021). Therefore, in this study, we directly tested the impact of exposure to heat stress on UPR^ER^ activation and identified a noncanonical HSF-1-dependent, but XBP-1-independent, UPR^ER^ in response to heat. In addition, we found that neural overexpression of *xbp-1s* previously found to promote whole-organism UPR^ER^ activation to extend lifespan (Taylor and Dillin 2013; Frakes et al. 2020) can similarly boost the HSR to promote thermotolerance to a comparable level as neuronal *hsf-1* overexpression (Douglas et al. 2015). Overall, our study adds further evidence to the complex interplay and general overlap between cellular stress signaling networks.

## Materials and methods

### *C. elegans* maintenance

All strains used in this study are derived from N2 wild-type worms and were previously constructed, validated, and published in previous studies. All reporter strains are available on the *Caenorhabditis* Genetics Center (CGC) as referenced in reagents, and transgenic animal strains will be made available upon request. All animals are maintained at 15 °C fed OP50 *Escherichia coli* B strain during maintenance. For experiments, animals are synchronized using a standard bleaching protocol as described previously (Torres et al. 2022). Briefly, worms are collected into a 15 mL conical tube using M9 solution (22 mM KH_2_PO_4_ monobasic, 42.3 mM NaHPO_4_, 85.6 mM NaCl, 1 mM MgSO_4_) and subjected to a bleaching solution (1.8% sodium hypochlorite, 0.375 M NaOH in M9) at a 20:1 bleach:worm volume ratio. Bleach:worm mixture is vigorously shaken until all warm carcasses are dissolved, and intact eggs are washed 4 times using M9 solution by centrifugation at 1,100 × *g* for 1 min. After the final wash, animals were L1 arrested by incubating overnight in 12 mL M9 solution at 20 °C on a rotator for a maximum of 24 h. After bleaching, worms were then plated onto HT115 *E. coli* K strain from L1 until day 1 adulthood where all experiments were performed. *C. elegans* strains available in Supplementary Table 1.

### Plates

Standard Nematode Growth Medium (NGM) plates for maintenance using OP50 contained the following: Bacto-Agar (Difco) 2% w/v, Bacto Peptone 0.25% w/v, NaCl_2_ 0.3% w/v, 1 mM CaCl_2_, 5 µg/mL cholesterol, 0.625 mM KPO_4_ pH 6.0, 1 mM MgSO_4_.

Standard NGM-RNAi plates for experimental conditions using HT115 bacteria contained the following: 2% RNAi plates for experiments contained the following—Bacto-Agar (Difco) 2% w/v, Bacto Peptone 0.25% w/v, NaCl_2_ 0.3% w/v, 1 mM CaCl_2_, 5 µg/mL cholesterol, 0.625 mM KPO_4_ pH 6.0, 1 mM MgSO_4_, 100 µg/mL carbenicillin, 1 mM IPTG.

### *C. elegans* microscopy

All imaging of reporter strains was performed as previously described (Bar-Ziv et al. 2020) at day 1 of adulthood using the synchronization method described above. For all RNAi experiments, animals were exposed to RNAi starting from the L1 stage. Specifically, the heat-shock reporter *hsp-16.2p::GFP* was imaged by performing heat-shock at 34 °C for 2 h in a heated incubator, followed by recovery at 20 °C for 2 h. Control animals were left at 20 °C during the duration of the experiment. For the UPR^ER^  *hsp-4p::GFP* and *hsp-6p::GFP* experiments, animals are heat-shocked at 34 °C for 2 h in a heated incubator, followed by recovery at 20 °C for 4 h.

For tunicamycin experiments, L4 animals are washed off plates and exposed to 25 ng/µL tunicamycin or 0.1% DMSO (control) for 4 h in M9 solution in a rotator at 20 °C. Tunicamycin or DMSO is washed 3× with M9, and animals are recovered on standard HT115 plates overnight (up to 16 h) and imaged at day 1 of adulthood (Bar-Ziv et al. 2020).

All imaging is performed by picking 10+ animals into a 5 μL pool of 100 mM sodium azide on a standard NGM plate without bacteria to induce paralysis. Once the sodium azide droplet dried, paralyzed animals were then lined alongside each other and imaged under a Leica M205FCA automated fluorescent stereomicroscope running LAS X software and equipped with a standard GFP filter, Leica LED3 light source, and Leica K5 camera. For all imaging experiments, 3 biological replicates were performed with 2 technical replicates each, and 1 representative image was chosen for use in figures. For quantification, Fiji (Schindelin et al. 2012) was used to draw a region of interest along each individual worm, and integrated density was measured and normalized to area. Graphing and statistical analysis were performed with GraphPad Prism 10 software using a Mann–Whitney test or Kruskal–Wallis test for multiple comparisons unless otherwise stated.

### Thermotolerance measurements

For thermotolerance experiments, animals were grown on RNAi plates on either empty vector (EV) or RNAi bacteria from the L1 stage. Day 1 adult animals were placed at 34 °C, and animals were scored for viability every 2 to 3 h until all animals were scored (Torres et al. 2022). All assays were performed on >3 biological replicates. Survival curves were plotted using GraphPad Prism 10 software, and statistics were performed using a log-rank test in GraphPad Prism 10.

### RNA-sequencing

RNA collection was performed on ∼800 animals at day 1 of adulthood. Animals were collected into TRIzol solution, and worms were freeze/thawed 3× with liquid nitrogen with a 30 s vortexing step between each freeze thaw cycle. After the final thaw, chloroform was added to a 1:5 chloroform/TRIzol ratio, and aqueous separation was performed using centrifugation in a heavy gel phase-lock tube (VWR, 10847-802). The aqueous phase was mixed 1:1 with isopropanol, and then RNA was extracted as per the manufacturer's directions using a Qiagen RNeasy Mini Kit (74106). Library preparation was performed using a Kapa Biosystems mRNA Hyper Prep Kit as per the manufacturer's protocol, and sequencing was performed at the Vincent J Coates Genomic Sequencing Core at the University of California, Berkeley using an Illumina HS4000 mode SR100. Three biological replicates were used per condition. Low-quality reads and adaptor sequences were trimmed using Trim Galore v0.6.7-1 (Krueger 2025). Reads were aligned to the WBcel235 genome using STAR-2.7.10a (Dobin et al. 2013), and a raw count matrix was generated using featureCounts from Subread v2.0.3 (Liao et al. 2019) and the WBcel235.107.gtf file. Differential gene expression was calculated with DESeq2 (Love et al. 2014), and sva v3.42.0 was used to correct for batch effects and nonbiological sources of variation (Leek et al. 2012). Gene ontology (GO) enrichment analysis was performed using enrichGO from clusterProfiler_4.14.6 (Yu 2024). Overrepresentation analysis of XBP-1 targets (Urano et al. 2002) found in our dataset was performed using compareCluster (fun = “enricher”) from clusterProfiler_4.14.6 (Yu 2024). Overrepresentation analysis of published *rab-3p::xbp-1s* data (Taylor and Dillin 2013) for differentially expressed HSF-1 targets and HSR genes (GO:0009408) was performed using compareCluster (fun = “enricher”) from clusterProfiler_4.14.6 (Yu 2024).

## Results

**Fig. 1.**
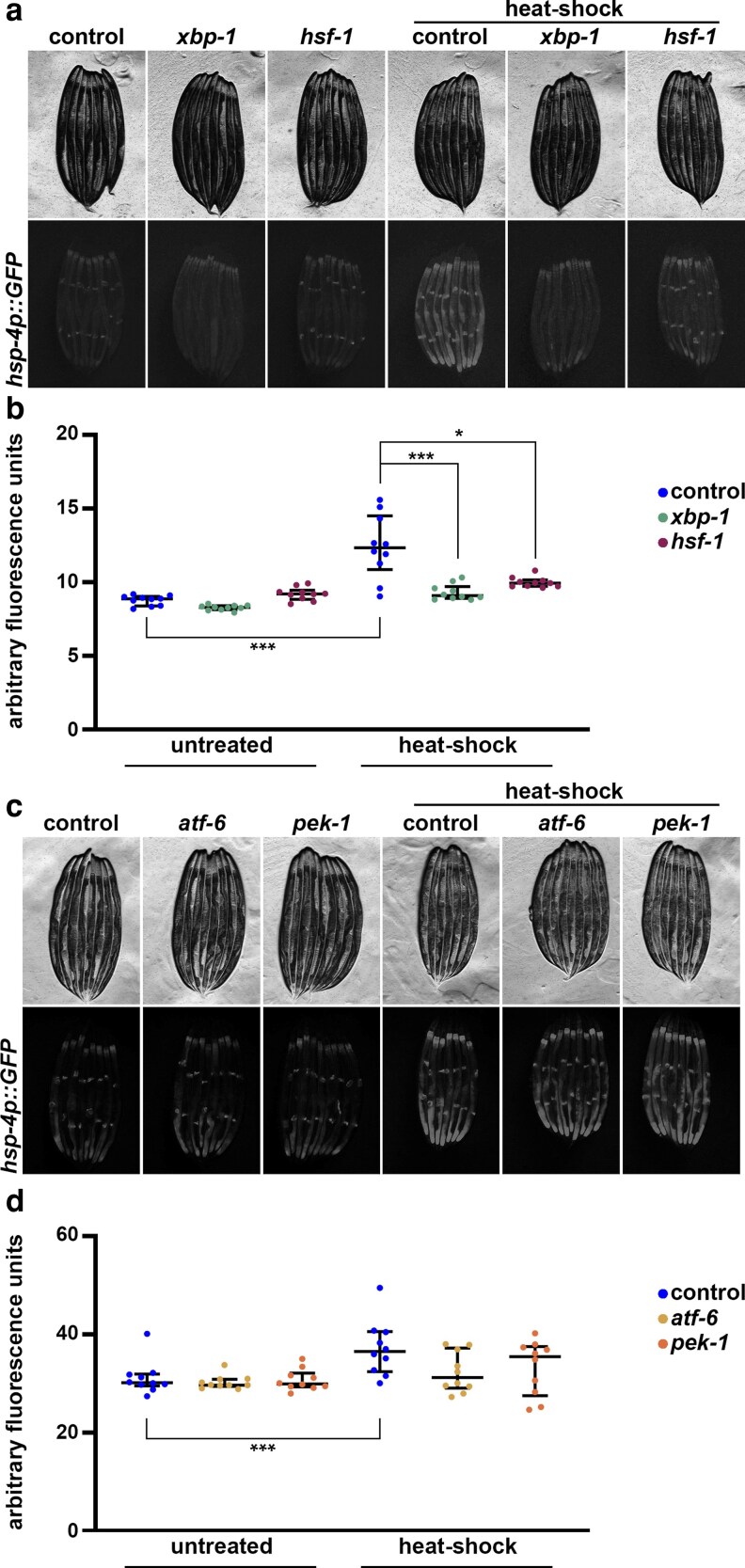
Heat stress induces UPR^ER^ in a *hsf-1* and *xbp-1* dependent manner. a) Representative fluorescent images of day 1 adult animals expressing *hsp-4p::GFP* grown on empty vector (EV, control), *hsf-1*, or *xbp-1* RNAi bacteria from L1. Heat-shock conditions are 2 h at 34 °C followed by a 4 h recovery at 20 °C. Data are representative of 3 independent replicates. b) Quantification of a represented as arbitrary fluorescent units, which are integrated fluorescence intensity measurements using ImageJ. Data are representative of 3 biological replicates where individual dots represent individual animals and lines represent median plus interquartile range. *P < 0.05; ***P < 0.001 using Kruskal–Wallis multiple comparison testing. c) Representative fluorescent images similar to a but grown on EV (control), *xbp-1*, *atf-6*, or *pek-1* RNAi bacteria. d) Quantification of c performed similar to b.

Previous studies have shown that heat-shock induces the UPR^ER^ in mammalian cells (Heldens et al. 2011). Here, we sought to determine whether heat-shock can also induce UPR^ER^ in vivo by testing the impact of heat-shock on the canonical UPR^ER^ reporter, *hsp-4p::GFP* in *C. elegans*, which is an indicator of *hsp-4*/HSPA5/BiP upregulation (Calfon et al. 2002). Indeed, the *hsp-4* promoter has predicted heat-shock responsive elements (Zha et al. 2019), suggesting potential activation of *hsp-4p::GFP* via HSF-1. We found significant activation of the *hsp-4p::GFP* reporter upon exposure to heat stress (Fig. 1a, b). Interestingly, this activation is dependent on both *hsf-1* and *xbp-1*, unlike UPR^ER^ induction via ER stress. Exposure to tunicamycin, which blocks n-linked glycosylation and induces misfolded protein stress in the ER (Heifetz et al. 1979), is dependent on *xbp-1*, but not on *hsf-1* (Supplementary Fig. 1). Importantly, the induction of heat-induced UPR^ER^ is not dependent on the other branches of the UPR^ER^, as *pek-1* or *atf-6* RNAi had no effect (Fig. 1c, d).

**Fig. 2.**
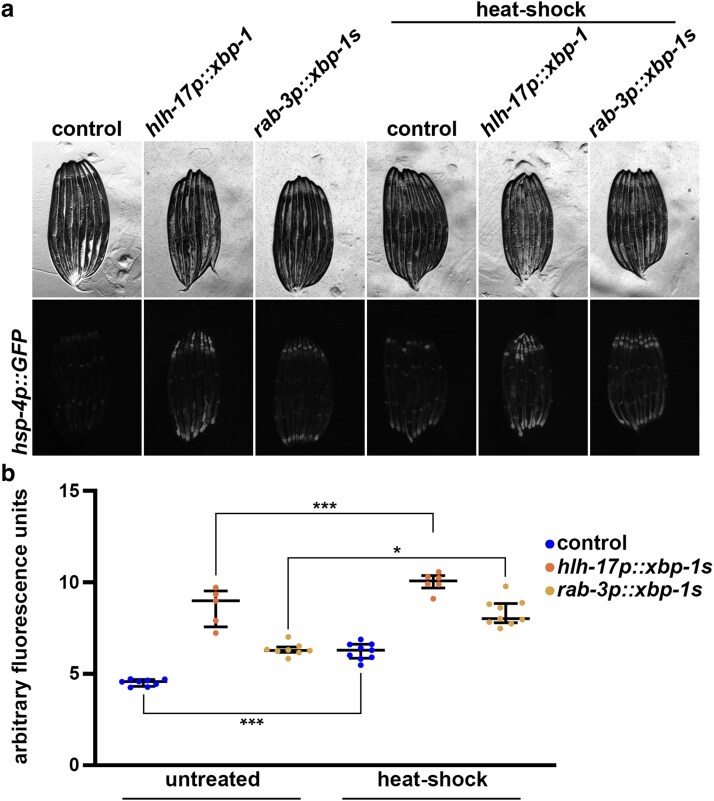
Neuronal or glial *xbp-1s* overexpression enhances heat-induced UPR^ER^. a) Representative fluorescent images of day 1 adult animals expressing *hsp-4p::GFP* alone or in combination with *hlh-17p::xbp-1s* or *rab-3p::xbp-1s* grown on EV (control) bacteria from L1. Heat-shock conditions are 2 h at 34 °C followed by a 4 h recovery at 20 °C. Animals are imaged on day 1 of adulthood. Data are representative of 3 independent replicates. b) Quantification of a represented as arbitrary fluorescent units, which are integrated fluorescence intensity measurements using ImageJ. Data are representative of 3 biological replicates where individual dots represent individual animals and lines represent median plus interquartile range. *P < 0.05; ***P < 0.001 using Kruskal–Wallis multiple comparison testing.

To determine whether the activation of the UPR^ER^ under heat stress was distinct from that activated under ER stress conditions, we simultaneously exposed *hsp-4p::GFP* animals to both heat and ER stress. To apply ER stress, animals were grown on RNAi against *tag-335*, a putative mannose-1-phosphate guanylyltransferase whose knockdown results in robust UPR^ER^ activity (Higuchi-Sanabria et al. 2020b). Interestingly, we found that *tag-335* knockdown and heat-shock did not have additive effects on UPR^ER^ activation, suggesting that a maximal capacity of UPR^ER^ activation is achieved upon *tag-335* knockdown (Supplementary Fig. 2). To further evaluate whether XBP-1 had any influence on heat-induced UPR^ER^, we next tested the impact of *xbp-1s* overexpression on UPR^ER^ activity. Consistent with previous reports, overexpression of *xbp-1s* in neuronal (Taylor and Dillin 2013; Daniele et al. 2020) or glial cells (Frakes et al. 2020) results in robust activation of UPR^ER^ (Fig. 2). Unlike UPR^ER^ activation via *tag-335* RNAi, UPR^ER^ induction via nonautonomous activation from glial or neuronal signals had additive effects with heat-induced UPR^ER^. These data suggest that either heat-induced or XBP-1-induced UPR^ER^ are distinct or that *xbp-1s* overexpression can enhance heat-induced UPR^ER^ activity.

**Fig. 3.**
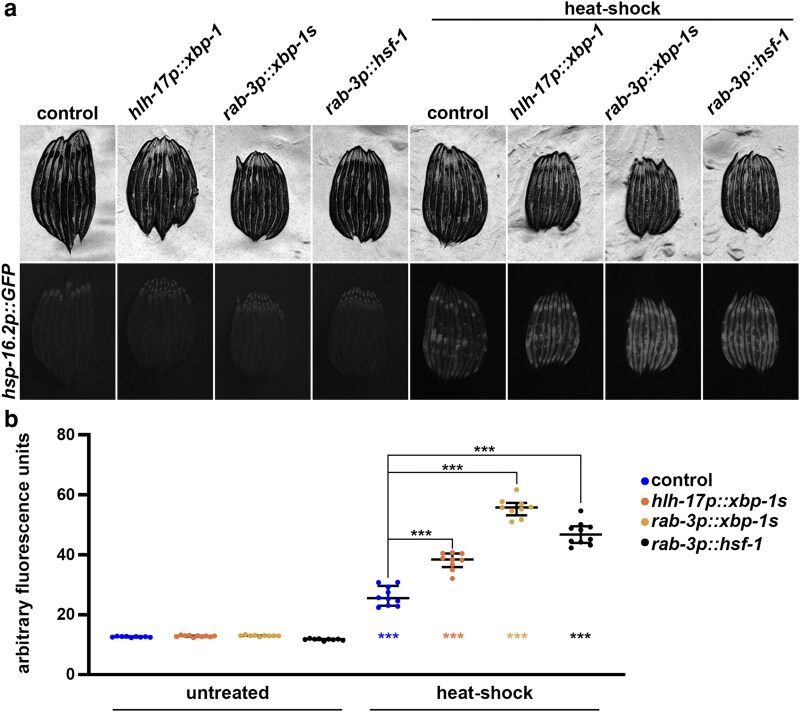
Neuronal and glial *xbp-1s* improves HSR similar to neuronal *hsf-1*. a) Representative fluorescent images of day 1 adult animals expressing *hsp-16.2p::GFP* alone or in combination with *hlh-17p::xbp-1s*, *rab-3p::xbp-1s*, or *rab-3p::hsf-1* grown on EV (control) bacteria from L1. Heat-shock conditions are 2 h at 34 °C followed by a 2 h recovery at 20 °C. Animals are imaged on day 1 of adulthood. Data are representative of 3 independent replicates. b) Quantification of a represented as arbitrary fluorescent units, which are integrated fluorescence intensity measurements using ImageJ. Data are representative of 3 biological replicates where individual dots represent individual animals and lines represent median plus interquartile range. ***P < 0.001 using Kruskal–Wallis multiple comparison testing. Colored *** below each plot indicates the *P*-value comparing heat-shocked animals vs their untreated control counterpart.

**Fig. 4.**
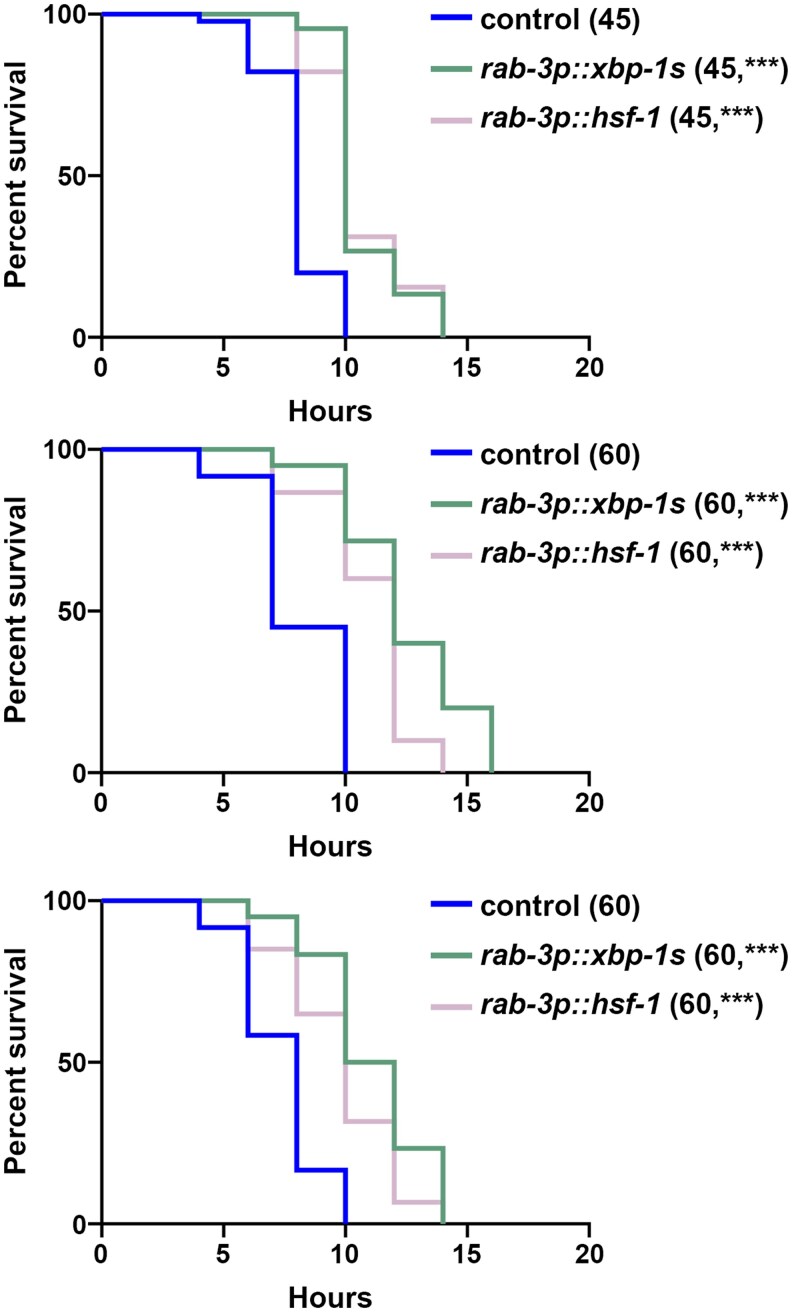
Neuronal *xbp-1s* improves thermotolerance similar to neuronal *hsf-1*. N2 wild-type (control), *rab-3p::xbp-1s*, or *rab-3p::hsf-1* animals grown on EV RNAi bacteria from L1. Animals were moved to 34 °C on day 1 of adulthood, and survival was scored every 2 h. All 3 biological replicates are shown for full transparency as thermotolerance measurements have been shown to display significant variability by several groups (Zevian and Yanowitz 2014; Bar-Ziv et al. 2020; Torres et al. 2022). Sample size is represented in the legend in parentheses, and ***P < 0.0001 vs control using log-rank testing in PRISM 10.

We next sought to determine whether regulators of UPR^ER^ could impact the activity of the HSR. To measure the HSR, we utilized the canonical HSR reporter, *hsp-16.2p::GFP*, which has a very low basal signal and robustly increases GFP expression upon exposure to heat stress (Link et al. 1999). To our surprise, we found that overexpression of *xbp-1s* in neurons or glial cells robustly enhanced the activity of the HSR, although it had no impact on basal *hsp-16.2p::GFP* expression (Fig. 3). Importantly, the increase in HSR induction upon exposure to heat stress is comparable to overexpression of *hsf-1* in neurons. This enhanced HSR by neuronal expression of *xbp-1s* was not affected by knockdown of *ire-1* or *xbp-1* but was completely eliminated upon *hsf-1* knockdown, similar to neuronal *hsf-1* animals, suggesting a sole requirement for *hsf-1* in this enhanced HSR (Supplementary Fig. 3). Finally, to determine whether this increase in HSR had any physiological impact, we next measured whether *xbp-1s* overexpression could improve thermotolerance, a canonical feature of *hsf-1* overexpression (Baird et al. 2014). Indeed, neuronal or glial *xbp-1s* overexpression displayed a significant increase in thermotolerance, again comparable to neuronal *hsf-1* overexpression, similar to its effect on the HSR (Fig. 4), suggesting this increased activation of the HSR is physiologically relevant.

Although the usage of transcriptional reporters allows for robust and rapid characterization of numerous conditions, it suffers from several caveats and limitations. First, GFP expression does not directly correlate with endogenous gene expression, as GFP is generally more stable than most RNA transcripts. In addition, all the transcriptional reporters presented here are overexpression constructs integrated in random gene loci, and thus the regulation of gene expression is likely not identical to the endogenous gene construct. Finally, the overreliance on a single gene reporter to define a complex stress response that involves dozens to hundreds of transcriptional changes can be inherently flawed. Indeed, a recent study (Kim et al. 2025) has shown that the overreliance on the *hsp-6p::GFP* URP^MT^ reporter may have been the direct cause of controversy of linking UPR^MT^ activity to longevity in *C. elegans* (Bennett et al. 2014). Therefore, to more globally understand the heat-induced UPR^ER^, we performed transcriptomic analysis.

Here, we performed RNA-sequencing on wild-type and neuronal *hsf-1* animals, with heat-shock (HS) and without treatment (UT). For neuronal *hsf-1*, we compared overexpression of full-length *hsf-1* (labeled FL for the transcriptomics section to avoid confusion) and a C-terminal deletion (CTD) of *hsf-1*, which has been shown to maintain its ability to activate targets of the actin cytoskeleton while losing its capacity to induce the HSR (Baird et al. 2014). We also performed RNA-sequencing on the *hsf-1(sy441)* mutant, which carries the same CTD, which allows interrogation of HSF-1 activity in the absence of canonical HSR activation. All libraries displayed similar Phred scores >35, suggesting there was no systemic bias favoring any single condition and high quality of our libraries (Supplementary Fig. 4a). Sequencing results mapped sufficient and consistent unique reads across all samples (Supplementary Fig. 4b). Each biological replicate shared similar expression profiles as indicated by similar Spearman correlation (Supplementary Fig. 4c) and close clustering using principal component analysis (Supplementary Fig. 4d). As expected, the untreated groups clustered closely together, as did the heat-shocked conditions. Overall, the differential gene expression distribution of all animals looked similar; however, the *hsf-1(sy441)* mutant animals had the largest amount of uniquely differentially expressed genes (DEGs) (Supplementary Fig. 5), suggesting that the HSR signature of *hsf-1(sy441)* animals may be distinct from a canonical HSR.

**Fig. 5.**
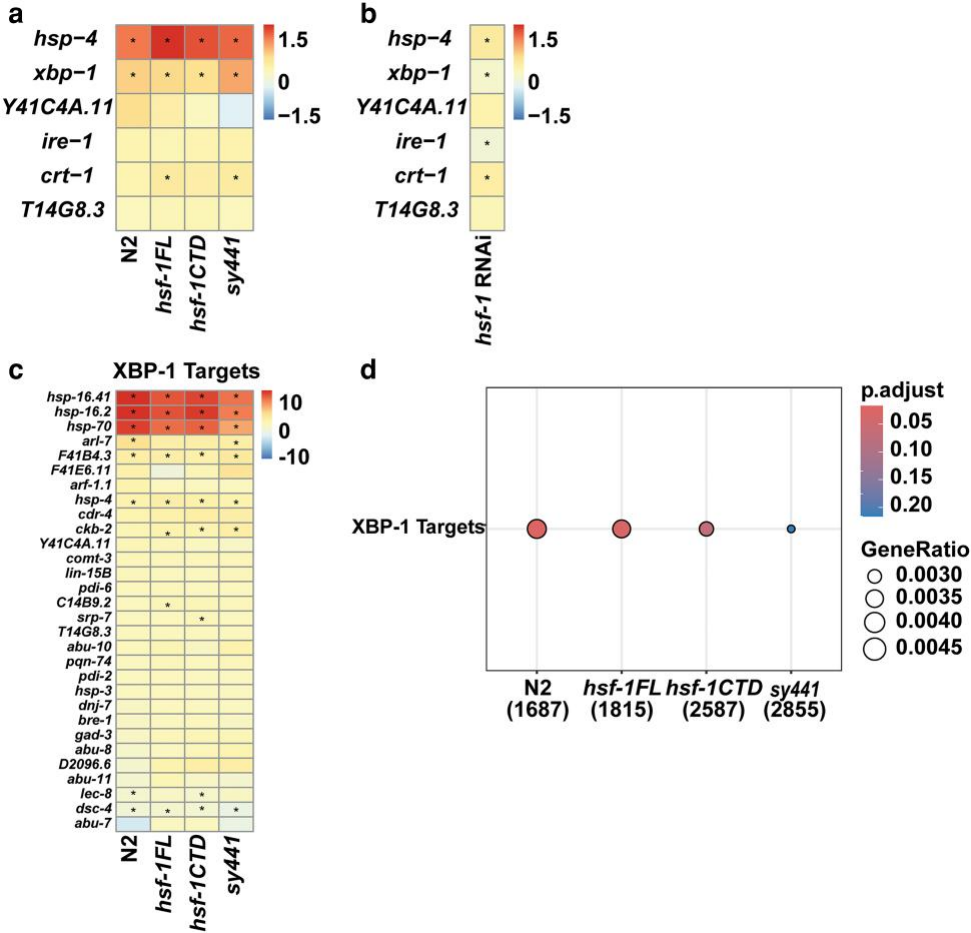
Heat-shock induces UPR^ER^ genes. RNA-sequencing was performed on N2, *rab-3p::hsf-1FL*, *rab-3p::hsf-1CTD*, and *hsf-1(sy441)* animals with and without heat-shock. DEGs were identified by comparing each heat-shock condition against the untreated and are available in Supplementary Table 2. A published RNA-seq dataset of N2 on *hsf-1* RNAi, with and without heat-shock, was reanalyzed with the same workflow (Brunquell et al. 2016). a) Heat-map of log_2_(fold-change) of N2, *rab-3p::hsf-1FL*, *rab-3p::hsf-1CTD*, *hsf-1(sy441)*, and *hsf-1* RNAi for canonical UPR^ER^ genes, *hsp-4*, *xbp-1*, *Y41C4A.11*, *ire-1*, *crt-1*, and *T14G8.3* upon heat-shock. Warmer colors indicate increased expression and cooler colors indicate decreased expression. *Adjusted *P*-value < 0.01. Values not *z*-score normalized. See Supplementary Table 3 for the gene list. b) Heat-map of log_2_(fold-change) of N2 on *hsf-1* RNAi for canonical UPR^ER^ genes, *hsp-4*, *xbp-1*, *Y41C4A.11*, *ire-1*, *crt-1*, and *T14G8.3* upon heat-shock. Warmer colors indicate increased expression and cooler colors indicate decreased expression. *Adjusted *P*-value < 0.01. Values not *z*-score normalized. See Supplementary Table 3 for the gene list. c) Heat-map of log_2_(fold-change) of XBP-1 targets upon heat-shock (Urano et al. 2002). Warmer colors indicate increased expression and cooler colors indicate decreased expression. *Adjusted *P*-value < 0.01. Values not *z*-score normalized. See Supplementary Table 3 for the gene list. d) Overrepresentation Analysis of differentially expressed XBP-1 targets from c found in our RNA-seq. Total number of differentially expressed genes stated in (values). Adjusted *P*-values: N2 = 0.012, *rab-3p::hsf-1FL* = 0.026, *rab-3p::hsf-1CTD* = 0.069, *hsf-1(sy441)* = 0.216.

Next, we utilized our RNA-seq dataset to validate our stress reporter findings. Consistent with our *hsp-4p::GFP* reporter data, wild-type animals treated with heat-shock resulted in a significant upregulation of *hsp-4* transcripts compared to their untreated controls (Fig. 5a, Supplementary Tables 2 and 3). Heat-shock also increased transcript levels of *xbp-1*, which provides further evidence that XBP-1-mediated UPR^ER^ is activated under conditions of heat-shock. Surprisingly, *hsf-1(sy441)* animals still displayed a similar increase in *hsp-4* transcripts and even more of an increase in *xbp-1* transcripts, suggesting that the canonical heat-shock response mediated by a full-length HSF-1 is not required for heat-induced UPR^ER^. To confirm this, we analyzed a previously published RNA-seq dataset of animals grown on *hsf-1* RNAi, with and without heat-shock (Brunquell et al. 2016), to determine if a true lack of *hsf-1* would impact the heat-induced UPR^ER^. Consistent with our reporter data, knockdown of *hsf-1* resulted in a severe reduction of *hsp-4*, *xbp-1*, and *ire-1* transcripts under heat stress, confirming *hsf-1* as essential for the heat-induced UPR^ER^ (Fig. 5b).

**Fig. 6.**
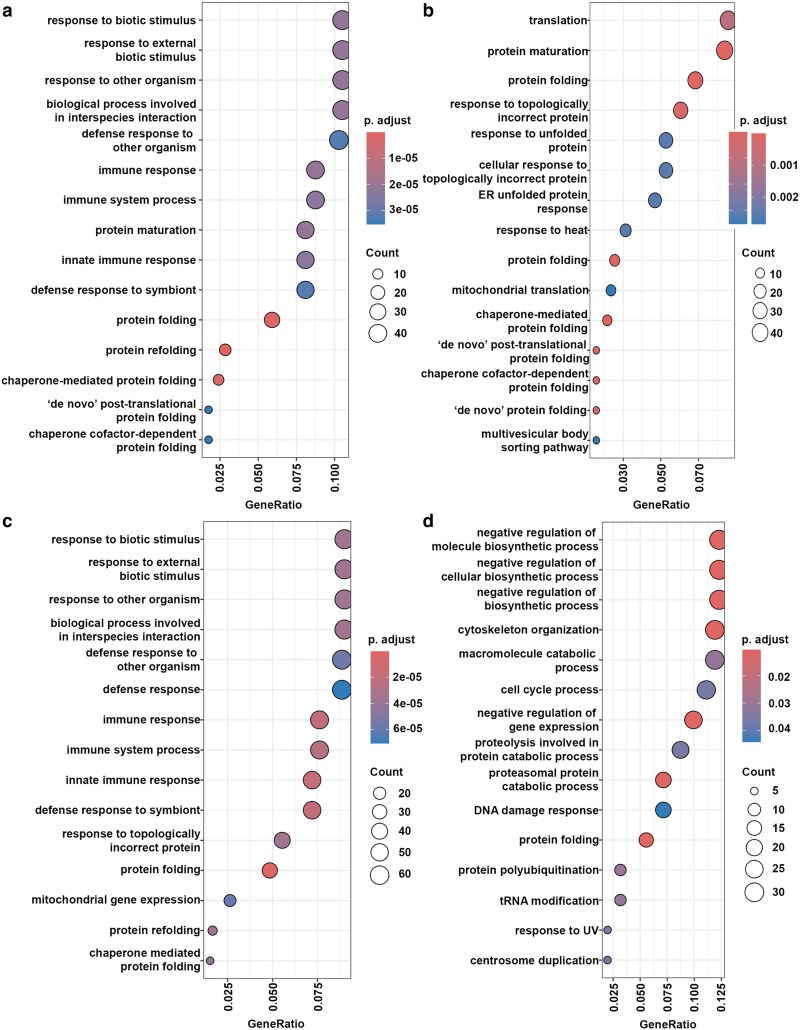
GO analysis of heat-shock induced genes. a) Top 15 GO terms for N2 heat-shock vs N2 untreated. b) Top 15 GO terms for *rab-3p::hsf-1FL* heat-shock vs *rab-3p::hsf-1FL* untreated. c) Top 15 GO terms for *rab-3p::hsf-1CTD* heat-shock vs *rab-3p::hsf-1CTD* untreated. d) Top 15 GO terms for *hsf-1(sy441)* heat-shock vs *hsf-1(sy441)* untreated. A table of all significant GO terms is available in Supplementary Table 5. All DEGs of each condition against N2 controls are also available in Supplementary Table 4.

Further analysis of our RNA-seq dataset revealed that at least a handful of XBP-1 targets (Urano et al. 2002) are differentially expressed under heat-shocked conditions (Fig. 5c, d). Importantly, gene ontology (GO) analysis of all significantly upregulated genes of heat-shocked vs untreated animals for each group showed an expected increase in genes associated with protein folding, chaperone function, and other HSR targets, which was lost in the *hsf-1(sy441)*, reproducing previous findings (Baird et al. 2014) (Fig. 6, Supplementary Table 4). Interestingly, the *hsf-1FL* included the UPR^ER^ as one of the top 15 enriched GO terms, and this GO term was significant in all conditions (Supplementary Table 5), providing further evidence that HSF-1 does indeed play an important role in the transcriptional activation of UPR^ER^ targets under heat stress. Indeed, upset plot analysis revealed that large similarities existed across all heat-shocked conditions (Supplementary Fig. 6a, b), with 372 shared downregulated DEGs and 392 shared upregulated DEGs. The common upregulated genes included expected biological processes downstream of HSF-1 activity, including immune response, protein refolding processes, and other processes involved with general proteostasis pathways. Importantly, the UPR^ER^ came up as a core shared gene ontology across all heat-shocked conditions (Supplementary Fig. 6c). Common downregulated genes included metabolic processes (Supplementary Fig. 6d), which are expected considering that animals tend to slow down metabolic processes in response to stress.

To further validate our bidirectional findings, we analyzed previously published transcriptomic data from neuronal *xbp-1s* animals (Taylor and Dillin 2013) and found that neuronal *xbp-1s* overexpression was also sufficient to induce a handful of HSF-1 and HSR target genes (Supplementary Fig. 7, Supplementary Table 6). These transcriptome analyses further substantiate the existence of a heat-induced UPR^ER^ that is likely dependent on both *xbp-1* and *hsf-1* and a possible overlap or co-regulation of genes between the UPR^ER^ and HSR.

## Discussion

Overall, our findings highlight the complex interplay between the HSR and the UPR^ER^ in *C. elegans*, emphasizing the existence of both compartment-specific and shared regulatory mechanisms. The observation that there exists a heat-induced HSF-1 and XBP-1 dependent UPR^ER^ distinct from the canonical UPR^ER^ mediated primarily by XBP-1 underscores the plasticity of stress response pathways. This adds to the growing literature of cross communication of stress responses, including prior work showing that cytosolic HSF-1 can influence mitochondrial proteostasis through fatty acid remodeling (Kim et al. 2016) and that mitochondrial stress can delay the age-associated loss of HSR activity (Labbadia et al. 2017). Similarly, our data suggest that cytosolic HSF-1 may directly—or indirectly—modulate ER stress response, although the exact mechanism of the transcriptional mechanism and shared chaperone networks remains to be elucidated. One interesting future avenue of research would be to understand the connection between HSF-1 and XBP-1. We found that heat-induced UPR^ER^ requires both transcription factors, but whether heat stress induces direct interaction of HSF-1 and XBP-1 or an indirect, coordinated effort to regulate transcription remains to be explored. Indeed, XBP-1 has been shown to interact with other stress response factors (Glover-Cutter et al. 2013), such as SKN-1, to regulate both ER and oxidative stress responses, suggesting interaction with other stress response transcription factors is possible.

The bidirectional cross talk between the UPR^ER^ and HSR adds complexity to the rapidly growing understanding of stress response integration. The integrated stress response (ISR) serves as a well-characterized framework for understanding the integration of multiple stress inputs into a shared response. The ISR responds to a diverse set of stressors including proteotoxic stress, nutrient deprivation, and organelle stress (Kalinin et al. 2023; Wek et al. 2023; Lu et al. 2024; Ryoo 2024). The ISR typically involves eIF2α phosphorylation via kinases like PEK-1 to reduce global translation and reduce translational burden under stress. Parallel to the reduction of translation via the ISR, our study highlights a convergent response to variable stressors to promote proteostasis. Our study finds that heat stress results in the induction of chaperones required for both cytosolic and ER function, akin to how mitochondrial stress preconditions the HSR (Labbadia et al. 2017), inhibition of cytosolic chaperones promotes activation of the UPR^MT^ (Kim et al. 2016), and loss of cytosolic proteostasis can be offset by mitochondrial proteostasis (Berendzen et al. 2016; Wang et al. 2024). These phenomena may reflect a general strategy wherein localized stress can activate numerous proteostasis pathways to prevent cellular damage.

This converging stress-responsive pathway is further strengthened through inter-tissue communication of stress. Our study adds to the growing understanding of nonautonomous signaling of stress by highlighting the ability of neuronal and glial *xbp-1s* overexpression to enhance HSR activity and thermotolerance, despite XBP-1 being dispensable for the HSR. This points to a heterotypic neuronal signaling mechanism. This mirrors earlier findings whereby neuronal *hsf-1* activates both HSF-1 and DAF-16 in peripheral tissues to activate both HSR and OxSR to extend longevity (Douglas et al. 2015). Whether neural *xbp-1s* similarly regulate a heterotypic response has not yet been explored, but neuronal *xbp-1s* has been shown to activate several distinct peripheral responses, including improved proteostasis (Taylor and Dillin 2013), lipid homeostasis (Imanikia et al. 2019b; Daniele et al. 2020), autophagy (Imanikia et al. 2019a), and immune response (Coakley et al. 2025). Similarly, glial *xbp-1s* also mediates a peripheral proteostasis (Frakes et al. 2020), lipid metabolism, and autophagy response (Metcalf et al. 2024). Importantly, both neuronal and glial *xbp-1s* lifespan extension required both peripheral XBP-1 and HLH-30 (Imanikia et al. 2019a; Metcalf et al. 2024), suggesting a similar heterotypic response. Our data suggest that neural *xbp-1s* also amplifies the HSR in the periphery to promote thermotolerance. Still to be determined is whether *hsf-1* is required for the other beneficial effects of neural *xbp-1s*, considering its previously established roles in proteostasis (Morley and Morimoto 2004; Hayashida et al. 2010), autophagy (Kumsta et al. 2017), lipid metabolism (Chauve et al. 2021; Watterson et al. 2022), and longevity (Hsu et al. 2003; Morley and Morimoto 2004).

In mammals, hypothalamic *Xbp1-s* activation improved hepatic metabolism, suggesting conserved principles of inter-tissue coordination (Williams et al. 2014). Whether these nonautonomous signals are also dependent on a heterotypic response including activation of HSF-1 or other transcriptional regulators is uncertain. However, human cancer cells activate an atypical UPR^ER^ activation upon exposure to heat stress, which was independent of canonical ER stress pathways like PERK (Heldens et al. 2011). Importantly, activated targets included ER chaperones, in particular HSPA5/BiP, the target of our *hsp-4p::GFP* reporter, suggesting relevance of our findings to a mammalian context. Overall, our work advances the understanding of the interconnectivity of stress responses and further substantiates *C. elegans* as a powerful in vivo model system for dissecting conserved mechanisms of systemic stress coordination. Future work is critical to focus on identifying the signaling mechanisms of the heterotypic responses of neural UPR to peripheral HSF-1 and understanding the potential interaction between XBP-1 and HSF-1 to coordinate both ER and heat stress responses to converge on shared downstream effectors to promote resilience and longevity.

## Supplementary Material

jkag141_Supplementary_Data

## Data Availability

All data are presented in the figures and tables of the manuscript. All RNA-sequencing data reanalyzed from previously published datasets and raw data are available at Annotare E-MTAB-9771 (Higuchi-Sanabria et al. 2020a) and the National Center for Biotechnology Information Sequence Read Archive repository accession number BioProject PRJNA589459 (Frakes et al. 2020). RNA-sequencing data produced here are available at the National Center for Biotechnology Information Sequence Read Archive repository accession number BioProject PRJNA1399626 and the National Center for Biotechnology Information Gene Expression Omnibus repository accession number GSE331181. Supplemental material available at G3 online.

## References

[jkag141-B1] Anckar J, Sistonen L. 2011. Regulation of HSF1 function in the heat stress response. Implications in Aging and Disease. 80:1089–1115. 10.1146/annurev-biochem-060809-095203. 21417720

[jkag141-B2] Baird NA et al 2014. HSF-1-mediated cytoskeletal integrity determines thermotolerance and life span. Science. 346:360–363. 10.1126/science.1253168. 25324391 PMC4403873

[jkag141-B3] Bar-Ziv R et al 2020. Measurements of physiological stress responses in *C. elegans*. J Vis Exp. 159:. 10.3791/61001. PMC7840273 32510480

[jkag141-B4] B’chir W et al 2013. The eIF2α/ATF4 pathway is essential for stress-induced autophagy gene expression. Nucleic Acids Res. 41:7683–7699. 10.1093/nar/gkt563. 23804767 PMC3763548

[jkag141-B5] Bennett CF et al 2014. Activation of the mitochondrial unfolded protein response does not predict longevity in *Caenorhabditis elegans*. Nat Commun. 5:3483. 10.1038/ncomms4483. 24662282 PMC3984390

[jkag141-B6] Berendzen KM et al 2016. Neuroendocrine coordination of mitochondrial stress signaling and proteostasis. Cell. 166:1553–1563.e10. 10.1016/j.cell.2016.08.042. 27610575 PMC5922979

[jkag141-B7] Brunquell J et al 2016. The genome-wide role of HSF-1 in the regulation of gene expression in *Caenorhabditis elegans*. BMC Genomics. 17:559. 10.1186/s12864-016-2837-5. 27496166 PMC4975890

[jkag141-B8] Caldwell SR, Hill KJ, Cooper AA. 2001. Degradation of endoplasmic reticulum (ER) quality control substrates requires transport between the ER and Golgi. J Biol Chem. 276:23296–23303. 10.1074/jbc.M102962200. 11316816

[jkag141-B9] Calfon M et al 2002. IRE1 couples endoplasmic reticulum load to secretory capacity by processing the XBP-1 mRNA. Nature. 415:92–96. 10.1038/415092a. 11780124

[jkag141-B10] Chauve L et al 2021. Neuronal HSF-1 coordinates the propagation of fat desaturation across tissues to enable adaptation to high temperatures in *C. elegans*. PLoS Biol. 19:e3001431. 10.1371/journal.pbio.3001431. 34723964 PMC8585009

[jkag141-B11] Coakley AJ et al 2025. Distinct responses to non-autonomous UPRER mediated by glutamatergic and octopaminergic neurons. Commun Biol. 8:1650. 10.1038/s42003-025-09036-1. 41286443 PMC12644781

[jkag141-B12] Dai C . 2018. The heat-shock, or HSF1-mediated proteotoxic stress, response in cancer: from proteomic stability to oncogenesis. Philos Trans R Soc Lond B Biol Sci. 373:20160525. 10.1098/rstb.2016.0525. 29203710 PMC5717525

[jkag141-B13] Daniele JR et al 2020. UPRER promotes lipophagy independent of chaperones to extend life span. Sci Adv. 6:eaaz1441. 10.1126/sciadv.aaz1441. 31911951 PMC6938708

[jkag141-B14] Dobin A et al 2013. STAR: ultrafast universal RNA-seq aligner. Bioinformatics. 29:15–21. 10.1093/bioinformatics/bts635. 23104886 PMC3530905

[jkag141-B15] Douglas PM et al 2015. Heterotypic signals from neural HSF-1 separate thermotolerance from longevity. Cell Rep. 12:1196–1204. 10.1016/j.celrep.2015.07.026. 26257177 PMC4889220

[jkag141-B16] Frakes AE et al 2020. Four glial cells regulate ER stress resistance and longevity via neuropeptide signaling in *C. elegans*. Science. 367:436–440. 10.1126/science.aaz6896. 31974253 PMC7357615

[jkag141-B17] Frakes AE, Dillin A. 2017. The UPR(ER): sensor and coordinator of organismal homeostasis. Mol Cell. 66:761–771. 10.1016/j.molcel.2017.05.031. 28622521

[jkag141-B18] Glover-Cutter KM, Lin S, Blackwell TK. 2013. Integration of the unfolded protein and oxidative stress responses through SKN-1/Nrf. PLoS Genet. 9:e1003701. 10.1371/journal.pgen.1003701. 24068940 PMC3772064

[jkag141-B19] Han J et al 2013. ER-stress-induced transcriptional regulation increases protein synthesis leading to cell death. Nat Cell Biol. 15:481–490. 10.1038/ncb2738. 23624402 PMC3692270

[jkag141-B20] Hayashida N et al 2010. Heat shock factor 1 ameliorates proteotoxicity in cooperation with the transcription factor NFAT. EMBO J. 29:3459–3469. 10.1038/emboj.2010.225. 20834230 PMC2964172

[jkag141-B21] Haze K et al 1999. Mammalian transcription factor ATF6 is synthesized as a transmembrane protein and activated by proteolysis in response to endoplasmic reticulum stress. Mol Biol Cell. 10:3787–3799. 10.1091/mbc.10.11.3787. 10564271 PMC25679

[jkag141-B22] Heifetz A, Keenan RW, Elbein AD. 1979. Mechanism of action of tunicamycin on the UDP-GlcNAc:dolichyl-phosphate Glc-NAc-1-phosphate transferase. Biochemistry. 18:2186–2192. 10.1021/bi00578a008. 444447

[jkag141-B23] Heldens L et al 2011. An atypical unfolded protein response in heat shocked cells. PLoS One. 6:e23512. 10.1371/journal.pone.0023512. 21853144 PMC3154502

[jkag141-B24] Higuchi-Sanabria R et al 2020a. Divergent nodes of non-autonomous UPRER signaling through serotonergic and dopaminergic neurons. Cell Rep. 33:108489. 10.1016/j.celrep.2020.108489. 33296657 PMC8820220

[jkag141-B25] Higuchi-Sanabria R et al 2020b. Lysosomal recycling of amino acids affects ER quality control. Sci Adv. 6:eaaz9805. 10.1126/sciadv.aaz9805. 32637599 PMC7319768

[jkag141-B26] Hsu A-L, Murphy CT, Kenyon C. 2003. Regulation of aging and age-related disease by DAF-16 and heat-shock factor. Science. 300:1142–1145. 10.1126/science.1083701. 12750521

[jkag141-B27] Imanikia S et al 2019a. Neuronal XBP-1 activates intestinal lysosomes to improve proteostasis in *C. elegans*. Curr Biol. 29:2322–2338.e7. 10.1016/j.cub.2019.06.031. 31303493 PMC6658570

[jkag141-B28] Imanikia S et al 2019b. XBP-1 remodels lipid metabolism to extend longevity. Cell Rep. 28:581–589.e4. 10.1016/j.celrep.2019.06.057. 31315038 PMC6656787

[jkag141-B29] Kalinin A, Zubkova E, Menshikov M. 2023. Integrated stress response (ISR) pathway: unraveling its role in cellular senescence. Int J Mol Sci. 24:17423. 10.3390/ijms242417423. 38139251 PMC10743681

[jkag141-B30] Kim H-E et al 2016. Lipid biosynthesis coordinates a mitochondrial-to-cytosolic stress response. Cell. 166:1539–1552.e16. 10.1016/j.cell.2016.08.027. 27610574 PMC5922983

[jkag141-B31] Kim J, Dutta N, Garcia G, Higuchi-Sanabria R. 2025 Apr 19. Transcriptomic analysis of mitohormesis associated with lifespan extension in *Caenorhabditis elegans*. GeroScience 10.1007/s11357-025-01912-2 [accessed 2025 Nov 4]. PMC13574753 41186664

[jkag141-B32] Kohno K et al 1993. The promoter region of the yeast KAR2 (BiP) gene contains a regulatory domain that responds to the presence of unfolded proteins in the endoplasmic reticulum. Mol Cell Biol. 13:877–890. 10.1128/mcb.13.2.877-890.1993. 8423809 PMC358971

[jkag141-B33] Krueger F . 2025. FelixKrueger/TrimGalore. [accessed 2025 Apr 13]. https://github.com/FelixKrueger/TrimGalore.

[jkag141-B34] Kumsta C, Chang JT, Schmalz J, Hansen M. 2017. Hormetic heat stress and HSF-1 induce autophagy to improve survival and proteostasis in *C. elegans*. Nat Commun. 8:14337. 10.1038/ncomms14337. 28198373 PMC5316864

[jkag141-B35] Labbadia J et al 2017. Mitochondrial stress restores the heat shock response and prevents proteostasis collapse during aging. Cell Rep. 21:1481–1494. 10.1016/j.celrep.2017.10.038. 29117555 PMC5726777

[jkag141-B36] Leek JT et al 2012. The sva package for removing batch effects and other unwanted variation in high-throughput experiments. Bioinformatics. 28:882–883. 10.1093/bioinformatics/bts034. 22257669 PMC3307112

[jkag141-B37] Liao Y, Smyth GK, Shi W. 2019. The R package Rsubread is easier, faster, cheaper and better for alignment and quantification of RNA sequencing reads. Nucleic Acids Res. 47:e47. 10.1093/nar/gkz114. 30783653 PMC6486549

[jkag141-B38] Link CD, Cypser JR, Johnson CJ, Johnson TE. 1999. Direct observation of stress response in *Caenorhabditis elegans* using a reporter transgene. Cell Stress Chaperones. 4:235–242. 10.1379/1466-1268(1999)004<0235:doosri>2.3.co;2 10590837 PMC312938

[jkag141-B39] Liu Y, Chang A. 2008. Heat shock response relieves ER stress. EMBO J. 27:1049–1059. 10.1038/emboj.2008.42. 18323774 PMC2323268

[jkag141-B40] Love MI, Huber W, Anders S. 2014. Moderated estimation of fold change and dispersion for RNA-seq data with DESeq2. Genome Biol. 15:550. 10.1186/s13059-014-0550-8. 25516281 PMC4302049

[jkag141-B41] Lu H, Koju N, Sheng R. 2024. Mammalian integrated stress responses in stressed organelles and their functions. Acta Pharmacol Sin. 45:1095–1114. 10.1038/s41401-023-01225-0. 38267546 PMC11130345

[jkag141-B42] Ma Y, Hendershot LM. 2003. Delineation of a negative feedback regulatory loop that controls protein translation during endoplasmic reticulum stress. J Biol Chem. 278:34864–34873. 10.1074/jbc.M301107200. 12840028

[jkag141-B43] Metcalf MG et al 2024. Cell non-autonomous control of autophagy and metabolism by glial cells. iScience. 27:109354. 10.1016/j.isci.2024.109354. 38500817 PMC10946330

[jkag141-B44] Morley JF, Morimoto RI. 2004. Regulation of longevity in *Caenorhabditis elegans* by heat shock factor and molecular chaperones. Mol Biol Cell. 15:657–664. 10.1091/mbc.E03-07-0532. 14668486 PMC329286

[jkag141-B45] Neef DW et al 2014. A direct regulatory interaction between chaperonin TRiC and stress-responsive transcription factor HSF1. Cell Rep. 9:955–966. 10.1016/j.celrep.2014.09.056. 25437552 PMC4488849

[jkag141-B46] Ryoo HD . 2024. The integrated stress response in metabolic adaptation. J Biol Chem. 300:107151. 10.1016/j.jbc.2024.107151. 38462161 PMC10998230

[jkag141-B47] Schindelin J et al 2012. Fiji: an open-source platform for biological-image analysis. Nat Methods. 9:676–682. 10.1038/nmeth.2019. 22743772 PMC3855844

[jkag141-B48] Schröder M, Kaufman RJ. 2005. The mammalian unfolded protein response. Annu Rev Biochem. 74:739–789. 10.1146/annurev.biochem.73.011303.074134. 15952902

[jkag141-B49] Shi Y, Mosser DD, Morimoto RI. 1998. Molecular chaperones as HSF1-specific transcriptional repressors. Genes Dev. 12:654–666. 10.1101/gad.12.5.654. 9499401 PMC316571

[jkag141-B50] Sural S et al 2020. HSB-1/HSF-1 pathway modulates histone H4 in mitochondria to control mtDNA transcription and longevity. Sci Adv. 6:eaaz4452. 10.1126/sciadv.aaz4452. 33087356 PMC7577724

[jkag141-B51] Taylor RC, Dillin A. 2013. XBP-1 is a cell-nonautonomous regulator of stress resistance and longevity. Cell. 153:1435–1447. 10.1016/j.cell.2013.05.042. 23791175 PMC4771415

[jkag141-B52] Taylor SKB et al 2021. *C. elegans* electrotaxis behavior is modulated by heat shock response and unfolded protein response signaling pathways. Sci Rep. 11:3115. 10.1038/s41598-021-82466-z. 33542359 PMC7862228

[jkag141-B53] Torres TC et al 2022. Surveying low-cost methods to measure lifespan and healthspan in *Caenorhabditis elegans*. J Vis Exp. 183:e64091. 10.3791/64091. PMC9881476 35665741

[jkag141-B54] Urano F et al 2002. A survival pathway for *Caenorhabditis elegans* with a blocked unfolded protein response. J Cell Biol. 158:639–646. 10.1083/jcb.200203086. 12186849 PMC2174003

[jkag141-B55] Walter P, Ron D. 2011. The unfolded protein response: from stress pathway to homeostatic regulation. Science. 334:1081–1086. 10.1126/science.1209038. 22116877

[jkag141-B56] Wang Y et al 2024. Metabolic regulation of misfolded protein import into mitochondria. Mapa K, Ron D, editors. eLife. 12:RP87518. 10.7554/eLife.87518. 38900507 PMC11189628

[jkag141-B57] Watterson A et al 2022. Loss of heat shock factor initiates intracellular lipid surveillance by actin destabilization. Cell Rep. 41:111493. 10.1016/j.celrep.2022.111493. 36261024 PMC9642076

[jkag141-B58] Wek RC, Anthony TG, Staschke KA. 2023. Surviving and adapting to stress: translational control and the integrated stress response. Antioxid Redox Signal. 39:351–373. 10.1089/ars.2022.0123. 36943285 PMC10443206

[jkag141-B59] Williams KW et al 2014. Xbp1s in Pomc neurons connects ER stress with energy balance and glucose homeostasis. Cell Metab. 20:471–482. 10.1016/j.cmet.2014.06.002. 25017942 PMC4186248

[jkag141-B60] Ye J et al 2000. ER stress induces cleavage of membrane-bound ATF6 by the same proteases that process SREBPs. Mol Cell. 6:1355–1364. 10.1016/s1097-2765(00)00133-7. 11163209

[jkag141-B61] Yu G . 2024. Thirteen years of clusterProfiler. Innovation. 5:100722. 10.1016/j.xinn.2024.100722. 39529960 PMC11551487

[jkag141-B62] Zevian SC, Yanowitz JL. 2014. Methodological considerations for heat shock of the nematode *Caenorhabditis elegans*. Methods. 68:450–457. 10.1016/j.ymeth.2014.04.015. 24780523 PMC4112136

[jkag141-B63] Zha J, Ying M, Alexander-Floyd J, Gidalevitz T. 2019. HSP-4/BiP expression in secretory cells is regulated by a developmental program and not by the unfolded protein response. PLoS Biol. 17:e3000196. 10.1371/journal.pbio.3000196. 30908491 PMC6448932

