# Supplementary material for: Heat stress reveals bidirectional cross talk between the heat shock response and UPRER in C. elegans
Source: G3 (Bethesda). 2026 Jun 22;16(8):jkag141. doi: 10.1093/g3journal/jkag141 (PMC13439955; doi:10.1093/g3journal/jkag141)
Supplement: Supplementary file 1 [file jkag141_supplementary_data.zip › Supplemental_Figures_and_Legends_G3-2026-406868.pdf]

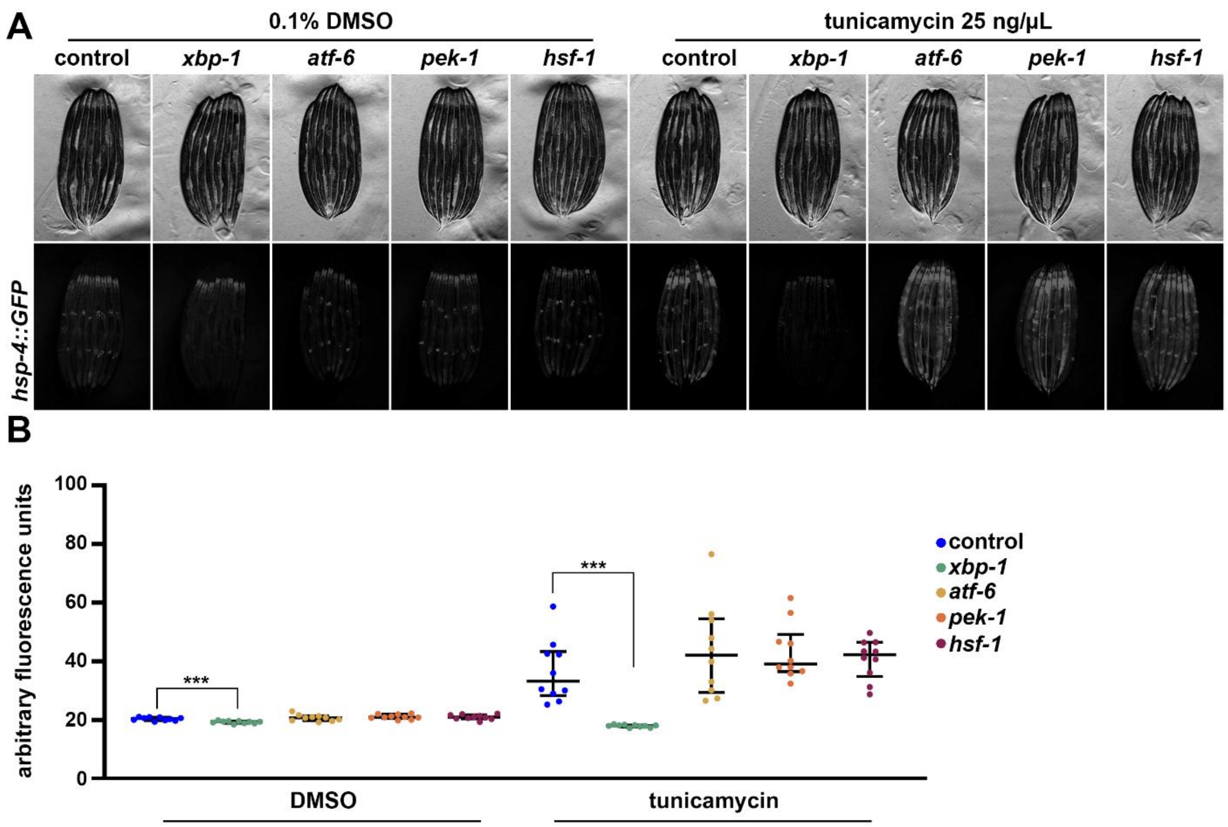

Fig. S1. ***hsf-1*** **is not required for ER stress induced UPR^{ER}. (A)** Representative fluorescent images of day 1 adult animals expressing *hsp-4p::GFP* grown on EV (control), *xbp-1, atf-6, pek-1,* or *hsf-1* RNAi bacteria from L1. L4 animals are treated with 0.1% DMSO or 25 ng/µL tunicamycin in M9 solution in a rotator at 20 °C for 4 hours. DMSO or tunicamycin are washed 3x with M9 solution and animals are placed on standard RNAi plates with EV RNAi bacteria and grown at 20 °C to recover overnight (maximum of 16 hours). Animals are imaged on day 1 of adulthood. Data is representative of 3 independent replicates. **(B)** Quantification of A represented as arbitrary fluorescent units, which are integrated fluorescence intensity measurements using Image J. Data is representative of three biological replicates where individual dots represent individual animals and lines represent median plus interquartile range. *** = $p < 0.001$ using Kruskal-Wallis multiple comparison testing.

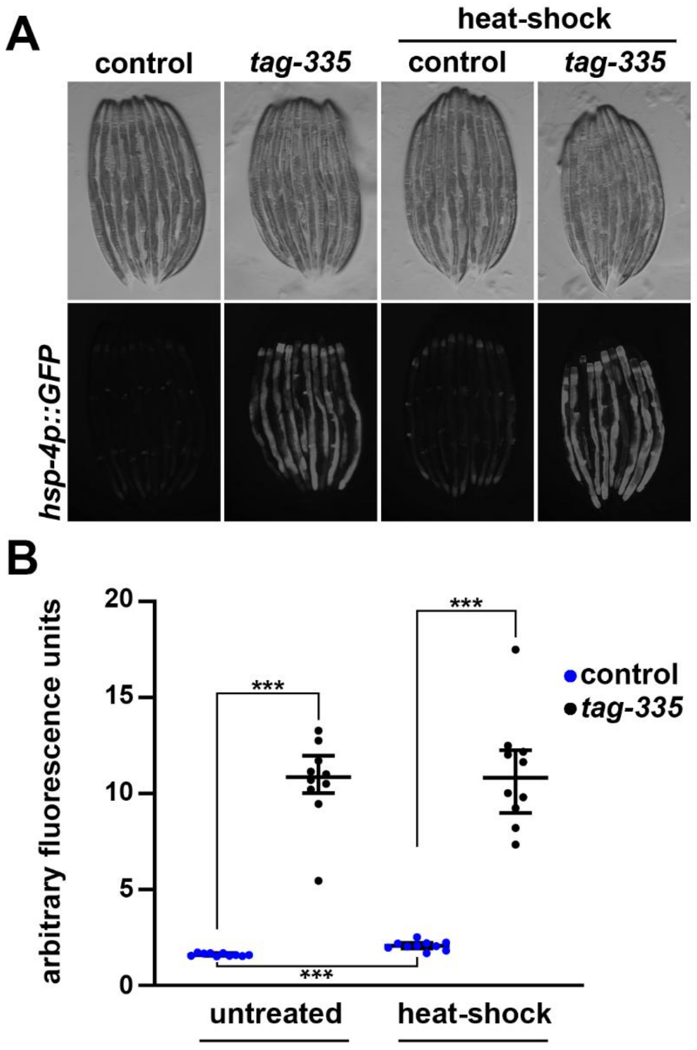

Fig. S2. ER and heat stress mediated UPR^{ER} are not additive. (A) Representative fluorescent images of day 1 adult animals expressing *hsp-4p::GFP* grown on EV (control) or *tag-335* RNAi bacteria from L1. Heat-shock conditions are 2 hours at 34 °C followed by a 4-hour recovery at 20 °C. Animals are imaged on day 1 of adulthood. Data is representative of 3 independent replicates. **(B)** Quantification of A represented as arbitrary fluorescent units, which are integrated fluorescence intensity measurements using Image J. Data is representative of three biological replicates where individual dots represent individual animals and lines represent median plus interquartile range. *** = $p < 0.001$ using Kruskal-Wallis multiple comparison testing.

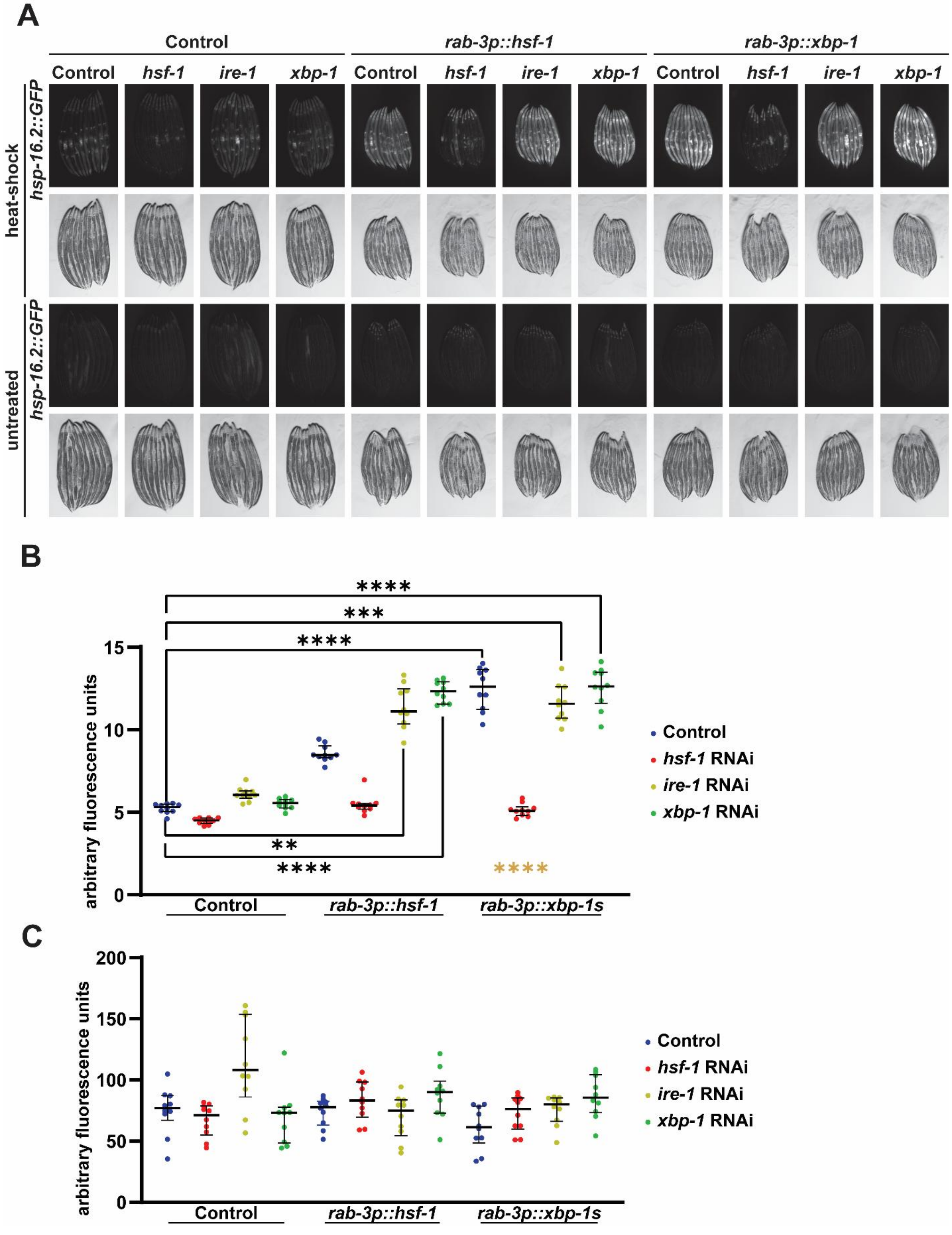
A
Control
rab-3p::hsf-1
rab-3p::xbp-1
Control
hsf-1
ire-1
xbp-1
heat-shock
untreated
hsp-16.2::GFP
B
****
***
****
**
****
****
arbitrary fluorescence units
15
10
5
0
Control
rab-3p::hsf-1
rab-3p::xbp-1s
Control
hsf-1 RNAi
ire-1 RNAi
xbp-1 RNAi
C
200
150
100
50
0
arbitrary fluorescence units
Control
rab-3p::hsf-1
rab-3p::xbp-1s
Control
hsf-1 RNAi
ire-1 RNAi
xbp-1 RNAi

Fig. S3. Neuronal *xbp-1s* enhancement of HSR is dependent on *hsf-1,* but not on *ire-1*. (A) Representative fluorescent images of day 1 adult animals expressing *hsp-16.2p::GFP* alone or in combination with *rab-3p::hsf-1* or *rab-3p::xbp-1s* grown on EV (control), *hsf-1*, *ire-1*, or *xbp-1* RNAi from L1. Heat-shock conditions are 2 hours at 34 °C followed by a 2-hour recovery at 20 °C. Animals are imaged on day 1 of adulthood. Data is representative of 3 independent replicates. **(B)** Quantification of heat-shocked samples from A represented as arbitrary fluorescent units, which are integrated fluorescence intensity measurements using Image J. Data is representative of three biological replicates where individual dots represent individual animals and lines represent median plus interquartile range. ** = $p < 0.01$, *** = $p < 0.001$,**** = $p < 0.0001$ using Kruskal-Wallis multiple comparison testing. Colored *** below each plot indicate p-value comparing heat-shocked animals vs. their control counterpart. **(C)** Quantification of untreated samples from A represented as arbitrary fluorescent units, which are integrated fluorescence intensity measurements using Image J. Data is representative of three biological replicates where individual dots represent individual animals and lines represent median plus interquartile range. No significant differences were found when compared to Control on EV using Kruskal-Wallis multiple comparison testing.

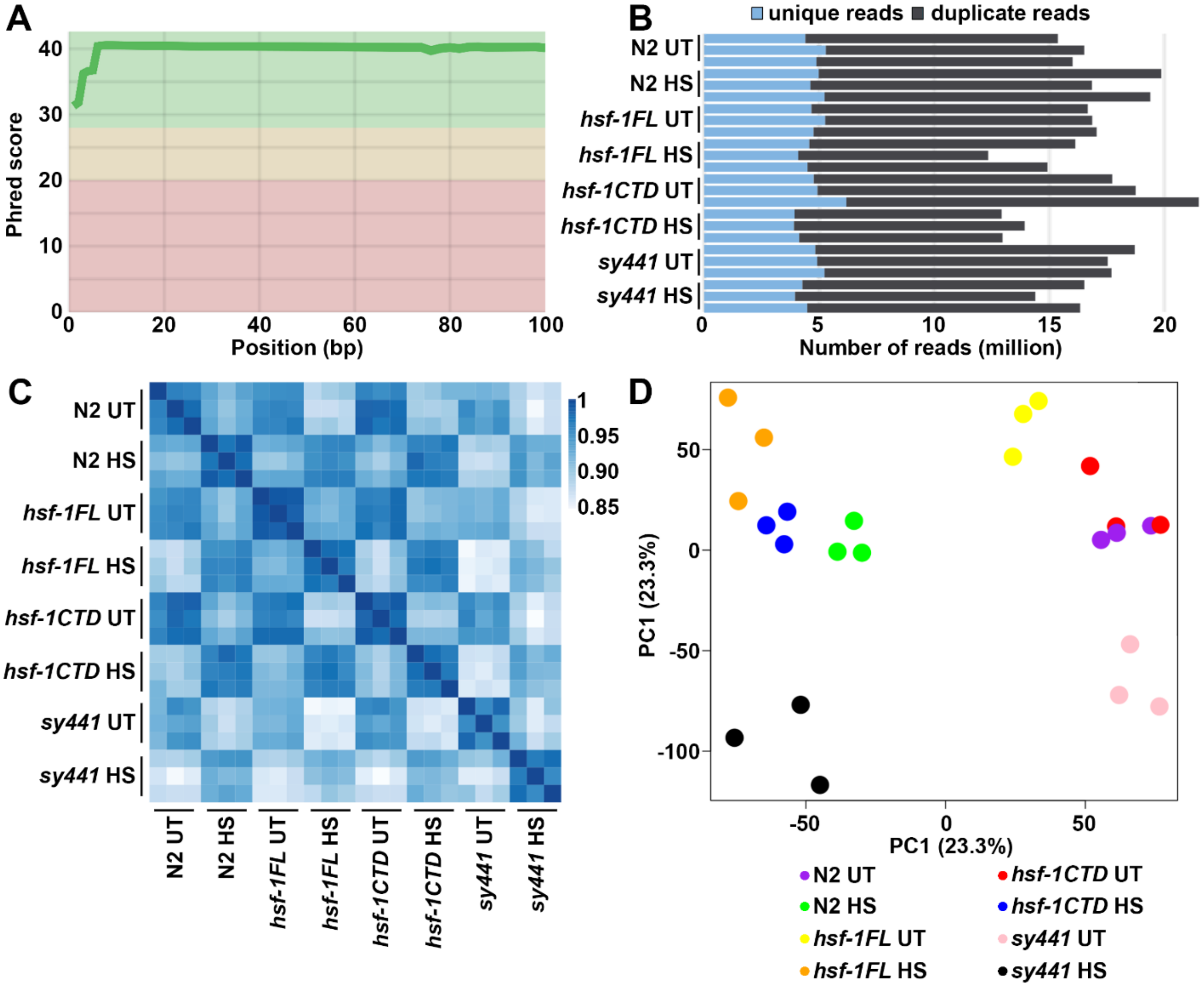

Fig. S4. Quality control for RNA-seq libraries of heat-shock activation in *C. elegans*. (A) Mean quality score (Phred score) of each RNA sequencing library. X-axis indicates the base

pair (bp) position of each sequence and the y-axis indicates the Phred score. The graph was generated by MultiQC(Ewels et al. 2016). **(B)** The number of unique (blue) and duplicated (black) reads from each pair-wise sequencing library (n = 3). **(C)** Spearman correlation plot of all RNA sequencing libraries (n = 3). **(D)** PCA plots of all RNA sequencing libraries.

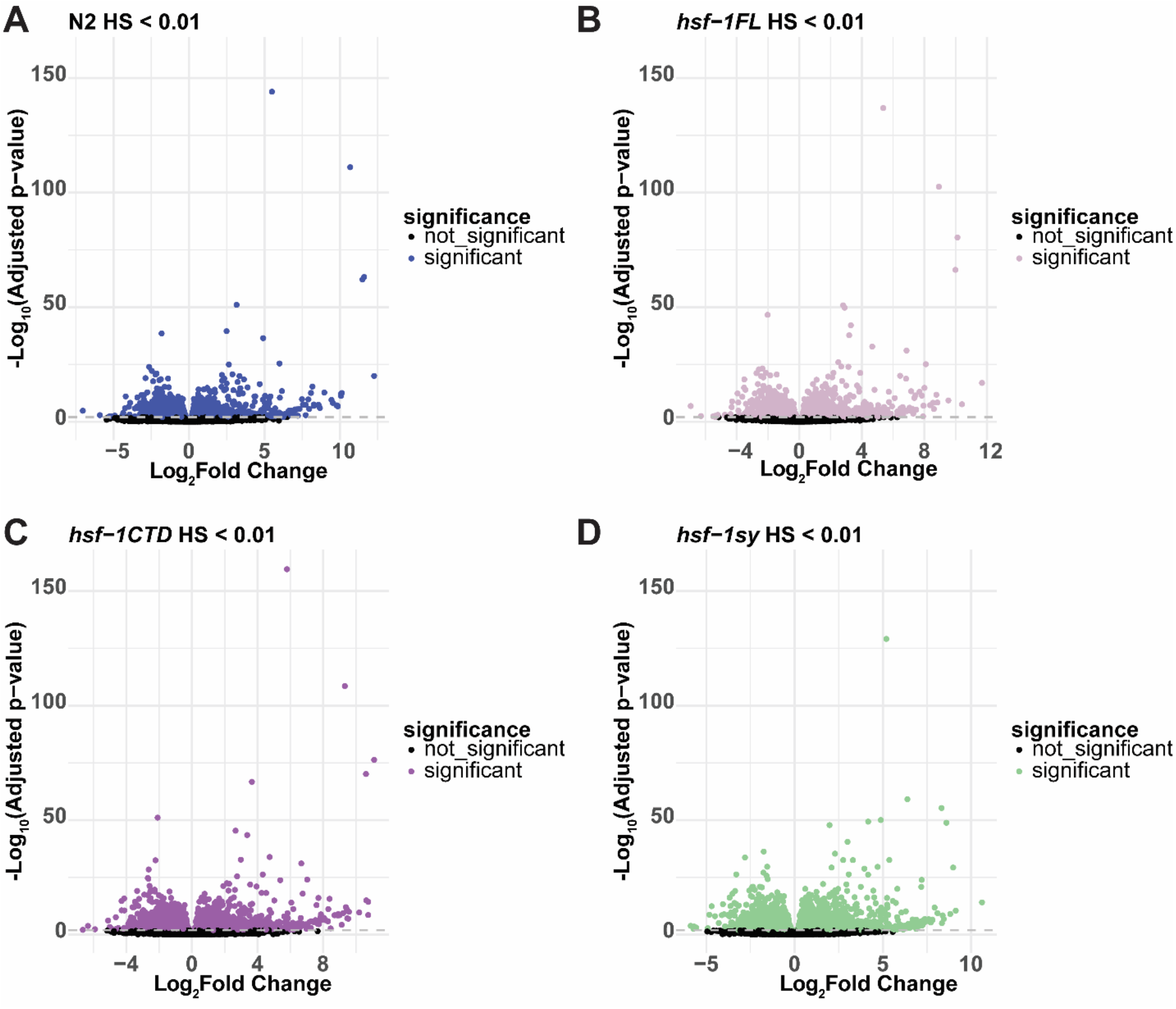

Fig. S5. Gene expression changes and comparison after heat-shock activation in *C. elegans*. Distribution of differentially expressed genes (p-value ≤ 0.01) in **(A)** N2, **(B)** *rab-3p::hsf-1FL*, **(C)** *rab-3p::hsf-1CTD*, and **(D)** *hsf-1(sy441)* animals after heat-shock. For a complete list of differentially expressed genes in each group, see **Table S3**.

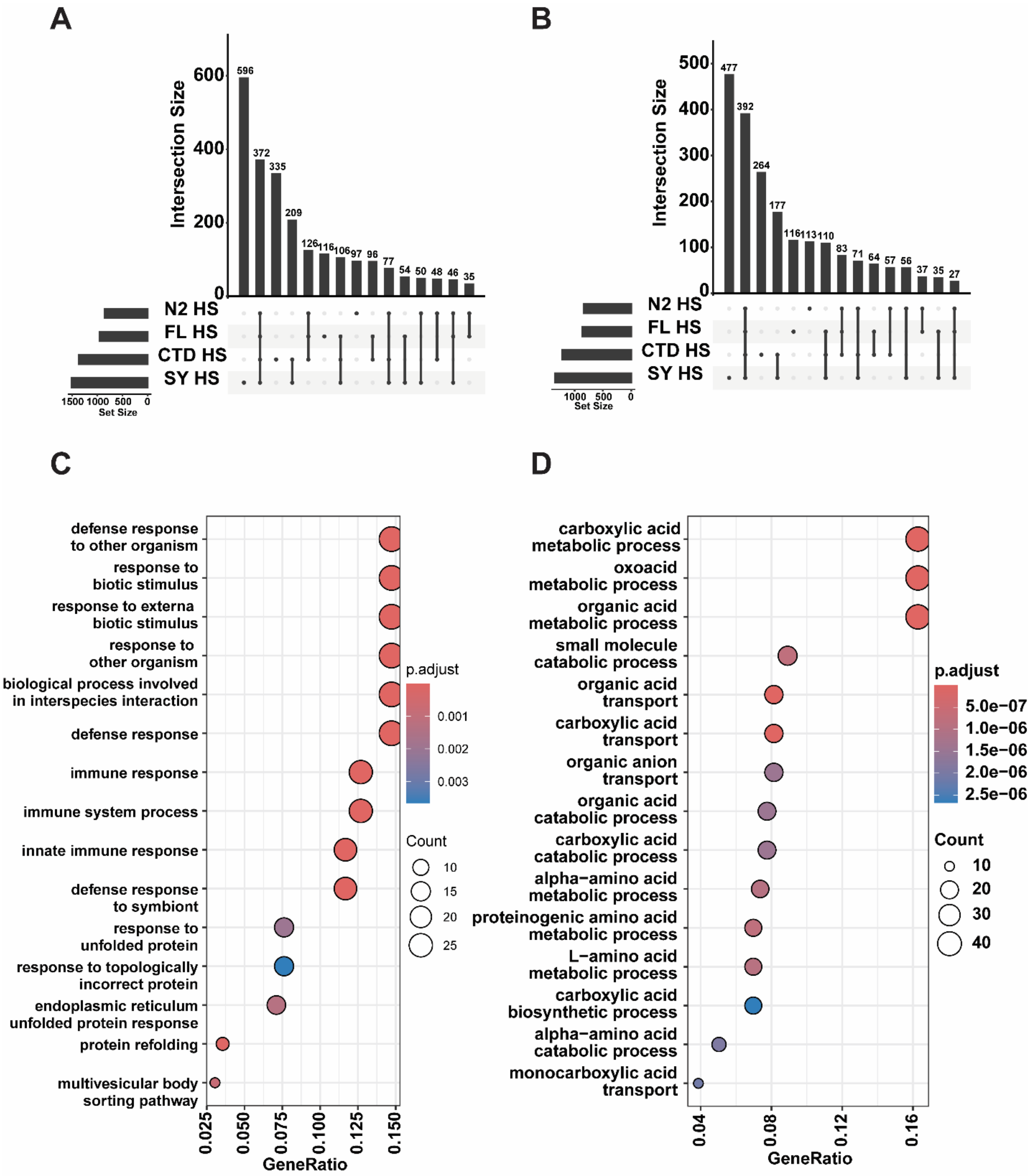

Fig. S6. Comparison of HSR signatures in *hsf-1* transgenic strains. (A) Upset plot comparing up-regulated N2, *rab-3p::hsf-1FL*, *rab-3p::hsf-1CTD*, and *hsf-1(sy441)* animal DEG sets after heat-shock. All the DEGs were determined by adjusted p-val < 0.01. **(B)** Upset plot comparing down-regulated N2, *rab-3p::hsf-1FL*, *rab-3p::hsf-1CTD*, and *hsf-1(sy441)* animal DEG sets after heat-shock. All the DEGs were determined by adjusted p-val < 0.01. **(C)** Top 15 GO-terms for all shared upregulated DEGs across all heat-shocked conditions (N2, *rab-3p::hsf-1FL*, *rab-3p::hsf-1CTD*, and *hsf-1(sy441)* animals). **(D)** Top 15 GO-terms for all shared

downregulated DEGs across all heat-shocked conditions (N2, *rab-3p::hsf-1FL*, *rab-3p::hsf-1CTD*, and *hsf-1(sy441)* animals).

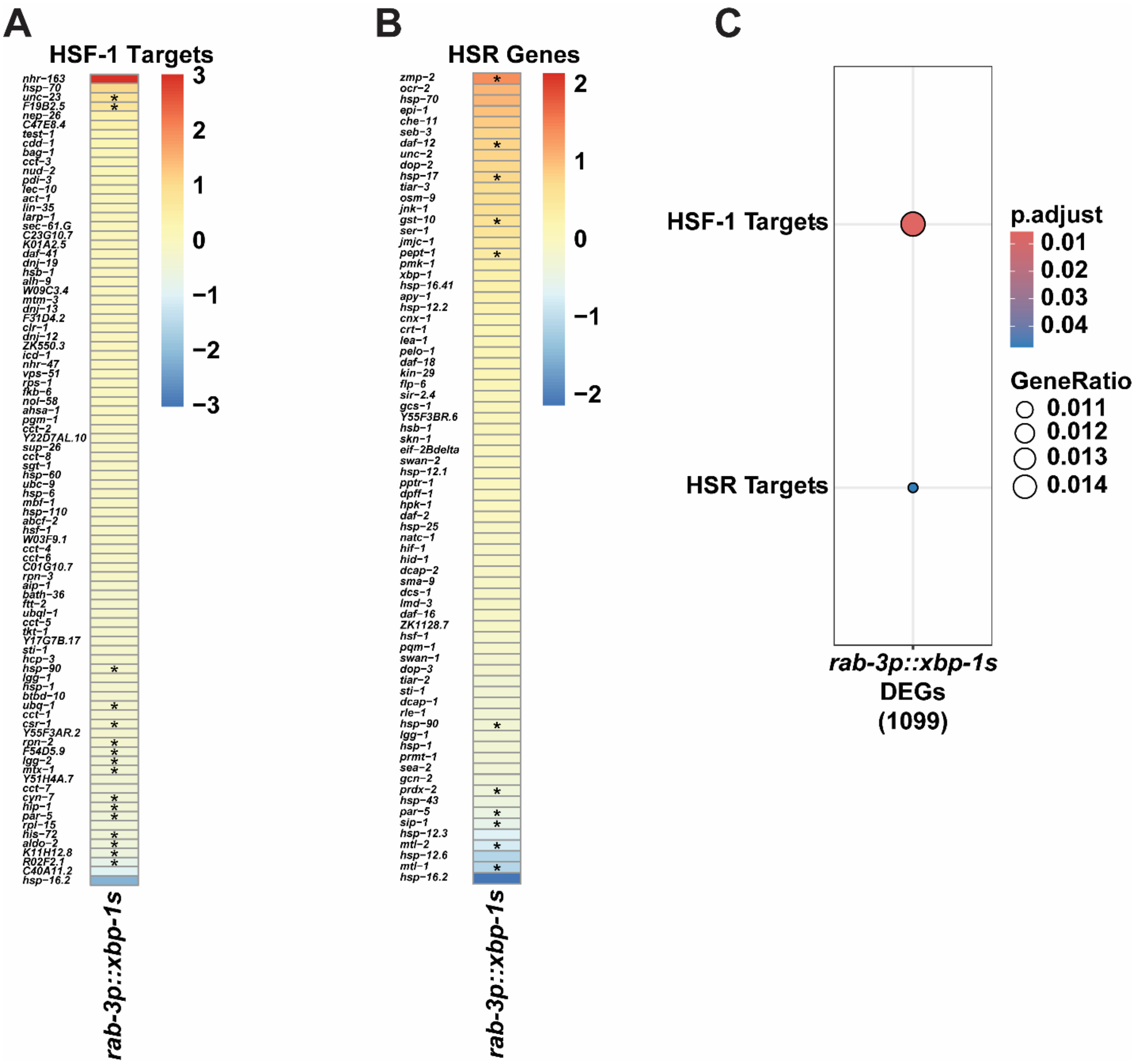

Fig. S7. Neuronal *xbp-1s* impacts HSF-1 targets and HSR genes. Analysis of a published RNAseq dataset from untreated *rab-3p::xbp1s* animals reanalyzed with the our workflow (Taylor and Dillin 2013). **(A)** Heat-map of $\log_2$(fold-change) of *rab-3p::xbp-1s* for HSF-1 target genes (Erinjeri et al. 2024). Warmer colors indicate increased expression and cooler colors indicate decreased expression. * = adjusted p-value < 0.01. Values not z-score normalized. **(B)** Heat-map of $\log_2$(fold-change) of *rab-3p::xbp-1s* for HSR genes (GO:0009408). Warmer colors indicate increased expression and cooler colors indicate decreased expression. * = adjusted p-value < 0.01. Values not z-score normalized. **(C)** Over-Representation Analysis of differentially expressed XBP-1 targets from **(A-B)** found in the dataset. Total number of differentially expression genes stated in (values). Adjusted p-values: HSF-1 targets=0.006, HSR genes=0.048. Gene lists are available in **Table S5**.

Table S1. Strains used in this study.

Table S2. DEGs of HS vs. UT.

Table S3. DEGs for UPR heat-map.

Table S4. DEGs against N2 controls.

Table S5. Table of all significant GO terms.

Table S6. DEGs for XBP-1 targets heat-map.
